# Assessing Changes in Anxiety, Empowerment, Stigma and Wellbeing in Participants Attending an Online-Based Recovery College in Quebec During the Covid-19 Pandemic: A Pre-Experimental Study

**DOI:** 10.3389/ijph.2022.1604735

**Published:** 2022-06-24

**Authors:** Filippo Rapisarda, Julio Macario de Medeiros, Catherine Briand, Antoine Boivin, Johana Monthuy-Blanc, Catherine Vallée, Marie-Josée Drolet, Brigitte Vachon, Francesca Luconi

**Affiliations:** ^1^ Centre de Recherche de l’Institut Universitaire en Santé Mentale de Montréal, Montréal, QC, Canada; ^2^ Département d’Ergothérapie, Université du Québec à Trois-Rivières, Trois-Rivières, QC, Canada; ^3^ Departement of Family Medicine, Centre de Recherche du CHUM, Centre hospitalier de l’Université de Montréal, Montréal, QC, Canada; ^4^ Groupe de Recherche Loricorps, Université du Québec à Trois-Rivières, Trois-Rivières, QC, Canada; ^5^ Department of Rehabilitation, Faculty of Medicine, Laval University, Québec, QC, Canada; ^6^ VITAM Research Centre on Sustainable Health, Laval University, Québec, QC, Canada; ^7^ Cervo Brain Research Centre, Laval University, Laval, QC, Canada; ^8^ École de Réadaptation, Faculté de Médecine, Université de Montréal, Montréal, QC, Canada; ^9^ Office for Continuing Professional Development, Faculty of Medicine and Health Sciences, McGill University, Montréal, QC, Canada

**Keywords:** anxiety, COVID 19 pandemic, program evaluation, mental health and wellbeing, recovery college, recovery, online-based

## Abstract

**Objectives:** The present study aims to evaluate the effect of an online Recovery College (RC) program implemented in Quebec (Canada) during the COVID-19 pandemic. From October 2020 to June 2021, 27 training groups were conducted with a total of 362 attendees.

**Methods:** Outcome was evaluated using a single group repeated measure design, assessing participants prior the training (T0), after the training (T1) and at follow up (T2). 107 learners of the Quebec RC program attended three two-hour sessions agreed to participate to the research.

**Results:** Overall findings show at T1 a small but statistically significant reduction of anxiety and increase in empowerment, and below threshold reduction of stigmatizing attitudes and increase of wellbeing. Conversely, the medium-term changes at follow up were non-significant for all the outcome dimension except for anxiety.

**Conclusion:** Findings suggest that the RC online program can be considered as a potential effective strategy to support self-regulation and empowerment of individuals and to reduce anxiety in the context of crisis for the general population.

## Introduction

The Covid-19 pandemic context (C-19) has a negative impact on the mental health of the global general population: health care providers, women, students, people with chronic conditions, and individuals with mental disorders were identified around the world as populations at risk for worsening of overall mental health during the pandemic [1–4]. To address these risks of mental deterioration and to strengthen protective factors, societies and health systems have had to deploy innovative public health strategies in prevention and promotion of mental health for all citizens, including health care providers and at-risk groups. Among these strategies, emphasis should be place on self-management approaches aimed at empowering individuals and communities to take action on their health and well-being [5, 6].

The C-19 has not only highlighted the vulnerabilities of individuals, but also those of our health care systems, which have been overwhelmed by the global burden of psychological needs and unable to fully address those needs through individual professional care, thus further widening health inequalities [7]. This has highlighted the importance of expanding health services beyond clinical and curative services to support public health actions in promoting mental health and preventing its deterioration, and thus, enabling individuals and communities to develop personal skills and find their own solutions in their living environments [8, 9]. The Recovery College (RC) model is one such strategy for mental health prevention and promotion [10]. Based on health education principles, the RC model is designed as a learning center offering a variety of trainings [11]. The challenge in the C-19 context was to quickly reach as many people as possible through online trainings.

### The Recovery College Model

Originally established in England in 2009, and now in 22 countries including Canada, the RC model offers a unique educational approach in the community where everyone may have access to trainings on well-being and mental health, empowerment, recovery and better living together [10, 11]. The Recovery College was developed within the recovery paradigm of mental health, a different way of thinking about the fundamental processes which underlie mental health care and services. The recovery paradigm, while not dismissing traditional symptom- and treatment- oriented interventions, goes beyond this by encouraging all people to develop their own understanding of their vulnerabilities and difficulties and to further develop self-management and other mutually supportive skills and resources [12]. The objectives of the RC trainings are to provide individuals with the opportunity to increase their personal skills, self-regulation and self-determination in mental health and well-being and to develop new knowledge that promotes individual and collective well-being and stigma reduction [12–15]. The model is based on the creation of a co-learning space where knowledge sharing between learners from various backgrounds are emphasized (e.g., individuals with or without mental health problems, relatives of health service users, mental health professionals, health and education service providers, teachers and university students, managers and employees of private organizations, committed citizens) [14–17]. The trainings offered in a RC are multiple, varied and co-constructed and co-facilitated by a dyad of trainers, a mental health worker and a person with experiential knowledge, that is, the lived experience of mental illness [11]. This approach aims to integrate in the trainings three type of knowledge: experiential knowledge, acquired through life experience as a person living with a mental illness, or as a family member or a caregiver of a person living with mental illness; clinical knowledge, acquired and applied by mental health practitioners and psychosocial group facilitators; theoretical knowledge, acquired through formal training in mental health (e.g., knowledge of evidence, scientific literature, theories) [10, 16, 17].

A narrative review of 31 peer reviewed articles [18] confirmed the positive outcomes of the RC model on a number of variables, including knowledge, self-regulation skills, empowerment, individual well-being, quality of life, recovery, reduced health care utilization, and recovery-oriented attitudes, beliefs and practices. These studies show significant change for in-person trainings of widely varying average duration (a few hours to several days) with the possibility of attending more than one training (high effect sizes between 0.78–0.86 in studies by Meddings [12, 15]). No studies have been conducted for short online trainings tailored to the C-19 context.

### Study’s Rationale and Scope

In autumn 2020, in Quebec, the C-19 pandemic emergency led to a new experimentation with online delivery of RC trainings, which has never been documented in the scientific or grey literature. Previous studies [12, 18] have primarily documented individual changes in wellbeing and empowerment among people with mental health problems attending traditional face-to-face RC trainings. Few studies have documented changes in the other types of learners considered in the RC model. The objective of the present study is to assess the outcomes of adapting the model to an online format in response to C-19 in order document its benefits on the general and at-risk population. The results of this first study will support the scaling up and generalization of the intervention and thus optimize the intervention to better meet the needs of the population.

## Methods

### Study Design

This pre-experimental study used a one-group pretest-posttest design with repeated measures. The baseline data collection (T0) took place one to 2 weeks prior to training participation during the registration process. The second data collection (T1) took place immediately after the end of the training sessions (after 1–2 weeks maximum). The third data collection (T2) took place 3 months after the end of the training sessions (after 12–15 weeks maximum). Data were collected during four training periods: two in autumn 2020 and two in spring 2021. According to the Quebec national public health institute (https://www.inspq.qc.ca/covid-19/donnees/ligne-du-temps), in the autumn of 2020, Quebec was under significant health measures and restrictions with an average of 1000 new cases per day (peak in December 2021 with an average of 2000 new cases per day). In the spring of 2021, half of Quebecers were vaccinated and the measures were being reduced. The number of new cases per day decreased from an average of 1500 new cases per day in April 2021 to an average of less than 500 new cases per day in May–June 2021.

### Recovery College in Quebec, Canada

At the onset of the pandemic, the Quebec RC converted its training from a traditional face-to-face format to an online format. Nine different trainings were developed, learners could choose the training according to their needs. Table 1 show the content topics of the nine different trainings. To rapidly reach as many people as possible and reduce accessibility barriers, the trainings were offered free of charge, over a period of 3 weeks, in three 2-h sessions, *via* Zoom.

**TABLE 1:** Online recovery college outline. Study: Outcome evaluation of on online based recovery college in Quebec (Canada, 2020–2021).

General principles and values
Based on educational principles
Based on co-production, co-learning, and close collaboration
Based on direct contact and diversity of learners to insure integration of knowledge
An integrated approach to community and social life
Social inclusion approach
Centred on the person and his learning process
Strengths and resources-based approach
A strategy that recognizes equity of knowledge and the contribution of experiential knowledge
**Available trainings**
Let’s talk about health, let’s talk about mental health in the context of pandemic
Towards well-being: dealing with stress
Resilience: adapting and equipping yourself to bounce back
Recovering motivation and meaning at work in a telework/tele-study context
Let’s discuss anxiety and worries
Performance anxiety in students: understanding it to better deal with it
Depression: together to better understand and cope with it
Schizophrenia without stigma: understanding the human before the disease
Recovery 101
Perspectives on stigmatization

From October 2020 until June 2021, 27 trainings were completed. Each training was attended by group of learners composed of 12–18 individuals, from different backgrounds, to ensure a diversity of perspectives, knowledge and expertise. To ensure a mix of backgrounds, invitations to register for trainings were sent out in collaboration with several partners in the health, education and community sectors, as well as through patient and family organizations, to reach populations at risk during the pandemic. Regardless of the training topic, each pair of certified trainers build an inclusive co-learning space for everyone to learn and share knowledge. To ensure a high level of interactivity between learners with different backgrounds, a variety of active teaching methods were used: teamwork exercises, group exchanges, experiential storytelling, short theoretical presentations, co-construction of online documents.

The reception of the online trainings has been positive. At the end of the two spring 2021 training periods, 95 learners out of 178 learners enrolled in the trainings agreed to respond to the satisfaction questionnaire. The majority of respondents (95%) were satisfied with the training they received and many (83%) had their expectations met. According to 94% of the participants who shared their opinion through the questionnaire, the format of the trainings offered opportunities for exchange and encouraged everyone to participate. In addition, despite the short time between the end of the trainings and the completion of the questionnaires, 84% of the respondents considered that what they learned during the training sessions was useful to them.

### Population and Recruitment Strategy

Recruitment was conducted through a convenience sampling procedure among all learners attending trainings. Potential participants received information about the study by email from the Quebec RC coordinator. In line with the RC model, minimal eligibility criteria were set, namely: being at least 16 years old, being able to attend an online meeting (in terms of technical equipment, computer skills and sensory and cognitive disabilities). Participation was entirely voluntary, and no incentives were offered. From a total number of 362 learners registered in the training groups that were invited in the study, 120 accepted to join the research project. For each measurement time, an online survey link was emailed, and targeted reminders were sent to participants who had not completed the survey.

### Measures

At each measurement time, data from each participant were collected through online survey. The survey included a first section collecting socio-demographic information such as gender, age, (formal) education level, mental health diagnosis across lifespan, type of knowledge on mental health (experiential, clinical and theoretical) and having received mental health care within the previous 6 months.

Validated instruments with good psychometric qualities were selected to assess changes over time. The selection of outcome dimensions was guided by previous studies for wellbeing, empowerment and stigma [18, 19]. The anxiety measure was added to address the C-19 pandemic context. Anxiety levels in the general population were a major public mental health issue during the C-19 pandemic [1–4, 20].

The Warwick and Edinburgh Mental Wellbeing Scale—Short Form (SWEMWBS) [21, 22] was chosen to assess mental well-being by covering a range of positive aspects. The Consumer Constructed Scale to Measure Empowerment (CCSME, a.k.a. Empowerment Scale) [23] assessed empowerment, defined empirically as a combination of self-efficacy, optimism and control over future, power, activism and righteous anger. The Opening Minds Stigma Scale for Health Care Providers (OMS-HC) [24] was developed to assess the impact of anti-stigma interventions. It evaluates people’s attitudes towards people with mental illness, attitudes toward disclosure and help-seeking and social distance. The Generalized Anxiety Disorder-7 questionnaire (GAD-7) [25], a seven-item-scale that assesses the frequency of anxiety symptoms within the past 2 weeks, was used as indicator of level of psychological distress. It has been used for assessing anxiety levels across different population studies [26] and as outcome measures for several clinical studies. Each item ranges from 0 to 4, a score of more than 8 corresponds to a clinical level of anxiety and more than 10 to a severe level of anxiety. In our study, internal consistency (Cronbach Alpha) was “good” for GAD7 (*α* = 0.87), OMS-HC (*α* = 0.81) and SWEMWBS (*α* = 0.80) and “acceptable” for CCSME (*α* = 0.77).

### Data Analysis

Descriptive statistics were computed for sociodemographic characteristics and questionnaires’ scores. Subjects that completed the assessment only at baseline without at least a second assessment at T1 or T2 were removed from the sample. Thirteen participants didn’t fill in the questionnaire at T1 and T2 and abandoned the research and were excluded from the analysis. Participants who didn’t fill questionnaires at T1 but did it at T0 and T2 were included in the study. No statistically significant differences were found at baseline between participants who were included and participants who were filtered off.

Changes over time were assessed using two different and complementary strategies. Statistically significant change of the intervention over T1 and T2 were tested using mixed linear models (MLM) for each outcome variable. MLM model included Time of data collection as a categorical variable (i.e., T0, T1 and T2) as predictor of changes and sociodemographic variables (age, gender, type of mental health knowledge, previous diagnosis and mental health care in the last 6 months) as fixed effect. The choice to include sociodemographic variables as fixed effects in the model was made to correct possible source of bias in estimating the Time effect caused by the dropout of participant or missing data at T1 and T2, and to explore possible association between outcome variables and individual characteristics.

Moreover, since data were collected during different phases of the Covid-19 pandemic, the month of data collection was considered a potential confounder that could bias the outcome evaluation. Thus, individual random intercepts and month of data collection as random effect to avoid the potential confounding effect of the pandemic and lockdown on individual changes. MLM analysis was done using the lme4 package v1.1-26 in R [27]. Magnitude of change was assessed computing Hedge’s *g* effect size.

The reliable change index (RCI) was computed for each outcome variable at T1 (compared to T0) and T2 (compared to T1) to estimate the number of participants that showed a significant increase or decrease. RCI is used to determine whether an estimation of true change over time has occurred when standardized by dividing by the standard error of measurement of the difference [28]. In the present study, the Maassen et al. formula was adopted [29].

### Ethical Consideration

This project has obtained ethical certification from two ethics committees, the Université du Québec à Trois-Rivières and the ethics committee of the Centre intégré universitaire de services sociaux et de santé de l’Est-de-l’Ile de Montréal (#MP-12-2021-2421). All procedures performed in studies involving human participants were in accordance with the ethical of the Centre intégré universitaire de services sociaux et de santé de l’Est-de-l’Ile de Montréal and with the 1964 Helsinki declaration and its later amendments or comparable ethical standards. All participants signed an information and consent form.

## Results

### Baseline Sample Descriptive Statistics

Sample sociodemographic information and baseline characteristics are reported in Table 2. Most of the participants were female (88%), with a mean age of 42.1 years and with an university degree. Participants’ background included: healthcare workers in public system (24.3%) or in non-profit organizations (7.5%), administrators, managers or supervisors working in the education or in the healthcare system (23%), college or university students (13.1%), person with lived experience of mental or physical illness (12.1%) or relatives (4.7%).

**TABLE 2:** Sample description at baseline. Study: Outcome evaluation of on online based recovery college in Quebec (Canada, 2020–2021).

	Total sample^a^
	N = 107
Gender	
Female	94 (87.9%)
Age	
Mean. sd	40.4 (14.7)
Education	
Professional course	6 (5.6%)
High school	6 (5.6%)
College	22 (20.6%)
University certificate	5 (4.7%)
Bachelor’s degree	33 (30.8%)
Masters’ degree	30 (28.0%)
Doctoral diploma	4 (3.7%)
Social role	
Healthcare workers from public system	26 (24.3%)
Administrative staff, manager, supervisor in educational or health system	25 (23.4%)
College or university student	14 (13.1%)
Person with lived experience of illness	13 (12.1%)
Healthcare workers from non-profit organization	8 (7.5%)
Relative of person with mental health or chronic physical disease	5 (4.7%)
Other citizen	16 (14.9%)
Mental health knowledge	
Experiential knowledge	61 (57.0%)
Clinical knowledge	41 (38.3%)
Theoretical knowledge	55 (51.4%)
Mental health parameters	
Received a diagnosis for a mental health problem lifetime	54 (50.5%)
Received mental health care in last 6 months	39 (36.4%)
Clinical level of anxiety^b^	36 (31.3%)
Severe level of anxiety^c^	21 (18.3%)
Possible depression^d^	2 (1.7%)
Season of attendance	
Fall 2020	47 (43.9%)
Spring 2021	60 (56.1%)
Training attendance	
Resiliency: adapting and equipping yourself to bounce back	19 (17.8%)
Perspectives over stigmatization	17 (15.9%)
Recovering motivation and meaning in remote working and studying	15 (14.0%)
Toward wellbeing, dealing with stress	14 (13.1%)
Let’s talk about anxiety and worry	13 (12.1%)
Let’s talk about health, let’s talk about mental health in the context of pandemic	13 (12.1%)
Schizophrenia without stigma: understanding the human before the disease	7 (6.5%)
Performance anxiety in youth	4 (3.7%)
Recovery 101	4 (3.7%)

^a^Nine participants were excluded from the analysis. They didn’t fill in the questionnaire at T1 and T2 or abandoned the research. No statistically significant differences were found at baseline between participant who were included and participants who were filtered off.

b Clinical level of anxiety ≥8 (GAD7).

c Severe level of anxiety ≥10 (GAD7).

d Possible depression <20 (WEMWS).

More than two thirds of participants stated to have an “experiential knowledge” of mental illness, one in two received a diagnosis of a mental health condition over the course of their lifetime and 36% received a mental health intervention during the last 6 months. At the same time, “theoretical knowledge” of mental illness and “clinical experience” were reported by, respectively, 51% and 38% of participants. Levels of clinical (GAD7 ≥ 8, as suggested by Plummer et al. [30]) or severe anxiety (GAD7 ≥ 10) in the sample were, respectively, 31.3% and 18.3%. However, the level of attendance varied across different trainings: lower levels of attendance was reported in those trainings focused on schizophrenia, performance anxiety in youth and recovery; conversely, higher attendance rates was reported for trainings that were related to coping skills (resiliency, dealing with stress, etc.) and stigma.

### Determinants of Outcome Measures

Statistically significant determinants of outcome variables were detected by the mixed models. Having received a diagnosis for a mental health problem was associated with higher GAD7 scores (*β* = 1.67; *p* < 0.05) and being a healthcare worker in the public system was associated with lower scores (*β* = −2.11; *p* < 0.05) compared to other social roles. No participants’ characteristics was associated with empowerment (CCSME) scores. For stigma (OMS-HC), the mixed model found a significant effect of age (*β* = 0.14; *p* < 0.05), and social roles, i.e., people with lived experience (*β* = −7.44; *p* < 0.01) and relatives (*β* = −8.58; *p* < 0.01) had lower levels of stigmatizing attitudes on total scores. For wellbeing (SWEMWBS), a significant effect was found for having a diagnosis of a mental health problem that was associated with lower scores (*β* = −0.58; *p* < 0.01).

### Outcome Evaluation

Tables 3, 4 report, respectively, changes in mean scores and categorical outcome evaluation for each outcome variable at T0, T1, and T2. For anxiety (GAD7), significant effect of Time found at T1 (*β* = −0.91; *p* < 0.01) and T2 (*β* = −0.076; *p* < 0.05), indicating a statistically significant and stable reduction in anxiety levels with a small effect size (0.23 at T1; 0.25 at T2). Congruously, the percentage of participants with a clinical level of anxiety shifted from 31.3% at baseline, to 19.6% at T1 and 22.0% at T2. Reliable change evaluation for anxiety detected at T1 a percentage of 10–12% of improvement (in term of significant reduction in comparison with baseline levels), and, at the same time, a smaller amount of worsening (2.3%), and the difference in percentage between the two outcomes was significant (X^2^ = 4.58; *p* < 0.05); however, at follow up, the difference in the distribution between improvement and deterioration was non-significant (X^2^ = 1.19; *p* = 0.27).

**TABLE 3:** Change in mean scores across time. Study: Outcome evaluation of on online based recovery college in Quebec (Canada, 2020–2021).

Outcome	Baseline (T0)	After RC (T1)		Follow up (T2)	
	Mean, sd	Mean, sd	Effect size^a^ (95%CI)	Mean, sd	Effect size^a^ (95%CI)
Anxiety (GAD7)	6.19 (4.25)	5.25 (3.75)**	0.23 (-0.04; 0.50)	5.15 (3.84)*	0.25 (-0.03; 0.54)
Empowerment (CCSME)	3.03 (0.24)	3.07 (0.26)*	0.16 (-0.11; 0.43)	3.08 (0.27)	0.20 (-0.09; 0.48)
Stigma (OMS-HC)	29.20 (7.22)	28.17 (6.94)	0.14 (-0.14; 0.43)	27.93 (6.21)	0.19 (-0.10; 0.47)
General Wellbeing (SWEMWBS)	25.77 (3.26)	26.35 (3.20)	0.18 (-0.09; 0.44)	26.31 (3.54)	0.16 (-0.12; 0.44)

GAD7, generalized anxiety disorder questionnaire; OMS-HC, opening minds stigma scale for health care providers; CCSME, consumer constructed scale to measure empowerment; SWEMWBS, Warwick-Edinburgh Mental Wellbeing Scale short form.

* = *p* < 0.05; ** = *p* < 0.01.

a Hedge’s *g* formula.

**TABLE 4:** Categorical outcome assessment with reliable change index of participants. Study: Outcome evaluation of on online based recovery college in Quebec (Canada, 2020–2021).

Scales	Reliable change T0 → T1			Reliable change T0 → T2		
	Estimated RCI	Participant improved N, %	Participants worsened N, %	Estimated RCI	Participant improved N, %	Participants worsened N, %
Anxiety (GAD7)^a^	4	11 (10.3%)	3 (2.3%)	4	13 (12.1%)	8 (7.5%)
Empowerment (CCSME)^b^	0.15	21 (19.6%)	8 (7.5%)	0.24	12 (11.2%)	8 (7.5%)
Stigma (OMS-HC)^a^	5	8 (7.5%)	5 (4.7%)	5	7 (6.5%)	6 (5.6%)
General Wellbeing (SWEMWBS)^b^	3	14 (13.1%)	10 (9.3%)	4	7 (6.5%)	11 (10.3%)

SWEMWBS, Warwick-Edinburgh Mental Wellbeing Scale short form; GAD7, generalized anxiety disorder questionnaire; OMS-HC, opening minds stigma scale for health care providers; Consumer Constructed Scale to Measure Empowerment.

a Improvement was evaluated as a significant reduction in scores.

b Improvement was evaluated as a significant increase in scores.

For empowerment (CCSME), statistically significant effect of time was found in the mixed model at T1 (*β* = 0.04; *p* < 0.05), that didn’t last at T2. Even though mean scores and effect size were higher at T2 (e.s. = 0.20) compared to T1 (0.16), the model didn’t detect a statistically significant change. However, the reliable change assessment indicated a significant difference in the balance between reliably improved (19.6%) at T1 compared to participants whose empowerment decreased (X^2^ = 4.86; *p* < 0.05); value that was reduced at T2 to 11.2%; however, at T2, the difference in the percentage between reliably increased and decreased was non-significant (X^2^ = 0.08; *p* = 0.37), indicating a balance in outcome.

For stigma (OMS-HC), the effect of Time at T1 was subthreshold ((*β* = −0. 81; *p* < 0.60). The small effect sizes between 0.14 and 0.19, indicated a small decrease of stigma. Reliable change indexes showed at relative balance between increase and reduction of stigmatizing attitudes at T1 (X^2^ = 0.31; *p* = 0.58).and T2 (X^2^ = 0.08; *p* = 0.78).

Finally, for wellbeing (SWEMWBS), a subthreshold effect was found for Time at T1 (*β* = 0.56; *p* < 0.61). Reliable change indexes indicated a relative balance between improved and worsened at T1 (X^2^ 0.66; *p* = 0.41) and T2 (X^2^ = 0.88; *p* = 0.34).

## Discussion

The purpose of the study was to evaluate the outcome of the online Quebec RC in reducing anxiety and stigma and in improving empowerment and wellbeing in a sample of adult citizens from various backgrounds during the C-19. Overall findings showed at T1 a small but statistically significant change in anxiety and empowerment, two variables that are related to proximal changes in emotional and cognitive appraisals, and below threshold changes for stigmatizing attitudes and wellbeing. Conversely, the medium-term outcome of the RC training (follow up) wasn’t statistically significant for all the outcome dimension except for anxiety, and descriptive examination of the ratio between reliably improved and worsened indicated a relative balance in percentages.

These results are not in line with the findings from previous studies that evaluated RC [12, 18] that reported significant improvement in wellbeing and empowerment. However, some possible explanations may be formulated. First, the type of delivery format (i.e., online), duration, and number of training groups attended by learners might explain the absence of change in empowerment and wellbeing. Although previous studies do not provide a detailed description of the duration and number of RC trainings that learners attended, the duration of the online RC evaluated in the present study could be considered too short to facilitate significant change in well-being and stigmatizing attitudes. In a setting other than C-19, learners could have chosen to attend more than one training. Regards to this postulate, it can hypothesize that wellbeing and stigmatizing attitudes are dimensions that may require longer term intervention and experiences to observe a significant change. Secondly, characteristics of participants at baseline may also differ from previous studies. In fact, compared to previous studies, our sample was composed of people with multiple different background roles (students, mental health workers, managers, teachers, individuals with lived experience of illness), closer to representing the general population, and was not limited to people recruited in a mental health setting. Thus, levels of empowerment, stigmatizing attitudes (OMS-HC) and wellbeing (SWEMWBS) are higher compared to previous studies in general population [24] or previous RC [23]. Third, in our study, 30% of them presented a clinical level of anxiety, which was the variable with the highest variance compared to the mean score, thus changes in this variable were more likely to be detected. At the same time, attendance was higher for trainings whose topic covered coping skills, so it could by hypothesised that, because of the overall rise of anxiety due to the C-19, reduction of anxiety was a relevant aim for many participants that motivated them to join the RC. Thus, although the online RC was not designed specifically with the objective of reducing anxiety levels, participant’s levels of anxiety decreased even though the pandemic context was characterized by high levels of social isolation, psychological distress, and low access to face-to-face psychosocial support. Similar levels in anxiety reduction were also found in an online based psychoeducational intervention to prevent anxiety in university students [31]. It should be noted that, among the proposed trainings, stress management techniques and psychoeducation about anxiety and worry were included. In addition, the lived experience of people that have been coping with anxiety and distress may have been shared among participants. This result may see coherent with findings from studies about sudden gains in individual and group therapy for anxiety disorders [32, 33] indicated that reduction in anxiety levels may occur in early stages of interventions as a consequence of non-specific treatment factors such the positive therapeutic alliance and team climate.

### Strengths, Limitations and Future Research

The results must be interpreted considering some limitations in the research design. First, the study described the outcome in the real-world setting, but without making a comparison with a control group its effectiveness cannot be evaluated. Second, examining the characteristics of the sample at baseline, one can note some elements that may reduce its representativeness, such as a very small number of men compared to women, an overall high level of education, and a lower level of anxiety than reported in the literature in Canada during the same period [34]. Compared to previous studies, this study presents some relevant strengths. First, with a sample size of 107 participants, it is one of the RC studies with the largest sample in the literature. Second, participants come from diverse backgrounds and have a wide variety of social roles (people diagnosed with mental disorders who are peer supporters, healthcare workers, college students, etc.) that make the study’s findings more generalizable and applicable within a population health framework. In fact, it is possible that, compared to previous RC, the transition to online has increased the accessibility of trainings and decreased some of the barriers, reported in the literature [35] that usually have prevented its use such as transport difficulties, personal/family problems, financial hardship, poor timetabling, work commitments and health problems. Fourth, the inclusion of anxiety as a parameter to assess outcome allowed for the observation of a significant and stable impact on a relevant aspect of population mental health, especially during the context of the pandemic in which data report an increase in anxiety in both the general population and healthcare workers.

The present study presented some insights that may be tested in future research. Reliable change statistics suggest that RC learners may vary in terms of individual changes: for example, a participant may have a significant decrease in anxiety while increasing his/her stigmatizing attitudes, and categorical outcome could be clustered. The RC model emphasises person centred approach to learning, thus, an exploratory analysis of individual trajectories or clusters could be addressed in future research. Moreover, an analysis of the process of change in terms of specific (e.g., training content) and non-specific mechanisms (e.g., online team climate) is required to depict relevant mechanism of action of RC interventions.

### Conclusion

The results suggest that RC online trainings can be considered as a potential strategy to support self-regulation and empowerment of individuals and to reduce anxiety in the context of crisis for the general population. That said, the results suggest that the benefits evolve differently according to the needs and characteristics of the participants which warrants other studies to confirm. From a cost/benefit point of view, considering that this intervention is easily accessible and has short duration (only 6 hours), these are very interesting results in a crisis intervention context where rapid intervention is required to support the mental health of a population and in person contact may increase the risk of contagion. A scaling up and generalization of the intervention to the whole population of Quebec (and of the Canadian Francophonie) is desired by all the partners involved in this online experimentation of RC trainings.

The RC model, which combines evidence-based knowledge and lived experience in setting up a co-learning space for mental health, must continue to be studied so that it can become a standard for health promotion actions to be implemented worldwide. The RC model provides the opportunity to influence several health determinants as well as on the recovery process of people living with mental health problems.
